# Beyond Rarity: Insights Into the Diagnosis and Histopathology of Breast Granular Cell Tumors

**DOI:** 10.7759/cureus.56774

**Published:** 2024-03-23

**Authors:** Chinedum Okafor, Jehan Abdulsattar, Adaugo Nwanguma

**Affiliations:** 1 Pathology, Louisiana State University Health System, Shreveport, USA; 2 Pathology and Translational Pathobiology, Louisiana State University Health Sciences Center, Shreveport, USA

**Keywords:** breast pathology, pathology, breast ihc, breast cancer, granular cell tumor, breast cancer biology

## Abstract

Breast granular cell tumors, which are benign and rare tumors of the breast, pose a diagnostic challenge due to their rarity and nuanced clinical presentations. This article explores a unique case of a 41-year-old female with a biopsy-confirmed granular cell tumor, shedding light on the intricacies involved in diagnosis. Rooted in a neuroectodermal origin, particularly Schwann cells, these tumors demand a multidimensional diagnostic approach for accurate identification. Despite their predominantly benign nature, malignant variants exist, necessitating a thorough histomorphology examination, supported by immunohistochemistry, for precise classification. This article contributes to our understanding of breast pathology and emphasizes the pivotal role of histopathology in unraveling complexities associated with granular cell tumors, reaffirming the importance of a comprehensive diagnostic approach.

## Introduction

Breast granular cell tumors (GCTs), mirroring their histological counterparts in various anatomical sites such as the tongue, represent a rare occurrence within the breast, accounting for approximately less than 10% of all GCTs [1]. The rarity of this lesion introduces a diagnostic intricacy, characterized by nuanced clinical, physical, and radiological presentations. This article meticulously examines a compelling case involving a 41-year-old female, offering both a biopsy-confirmed GCT diagnosis and intriguing radiologic findings.

Despite diverse etiological hypotheses, a prevailing consensus attributes the origin of these tumors to a neuroectodermal lineage, specifically tracing back to Schwann cells [2]. The exploration of such cases not only enriches our comprehension of breast pathology but also underscores the need for a multidimensional diagnostic approach.

While the majority of GCTs exhibit a benign course, the literature contains reports of malignant and atypical variants distinguished by unique histomorphological features and criteria [2]. To conclusively identify and characterize these lesions, a thorough histomorphological examination, complemented by immunohistochemistry (IHC) when necessary, stands as the gold standard. This comprehensive diagnostic approach not only affirms the nature of GCTs but also guides precise clinical management, emphasizing the pivotal role of histopathology in unraveling the intricacies of this rare breast lesion.

## Case presentation

We present the case of a 41-year-old female referred to our patient clinic due to a biopsy-confirmed right breast GCT, alongside a second breast mass that was determined to be a benign papilloma. Despite undergoing a breast lumpectomy, the patient continued to experience numbness in the affected breast.

An ultrasound of the breast unveiled an indeterminate right subareolar mixed cystic and solid mass situated at the 12 o'clock position, measuring 0.9 x 0.8 x 0.7 cm, with an overall Bi-rads score of 4. Subsequent excisional biopsy with clear margin revealed a discrete, well-defined white nodular lesion.

**Figure 1:**
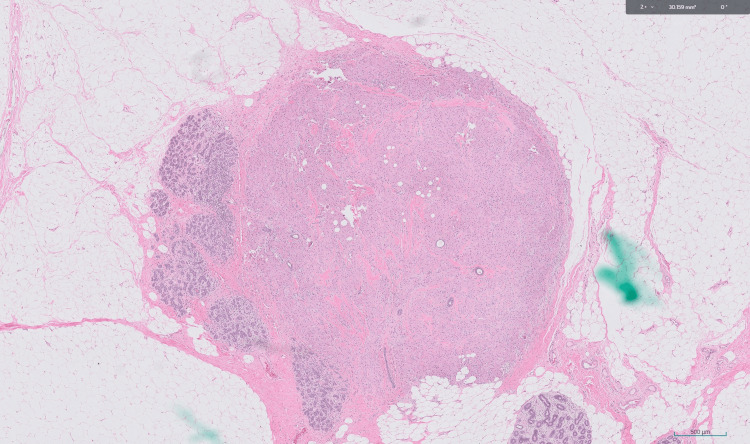
Granular cell tumor (x20 magnification) Microscopic examination revealed polygonal tumor cells with round nuclei and eosinophilic granular cells occurring next to benign breast lobules.

**Figure 2:**
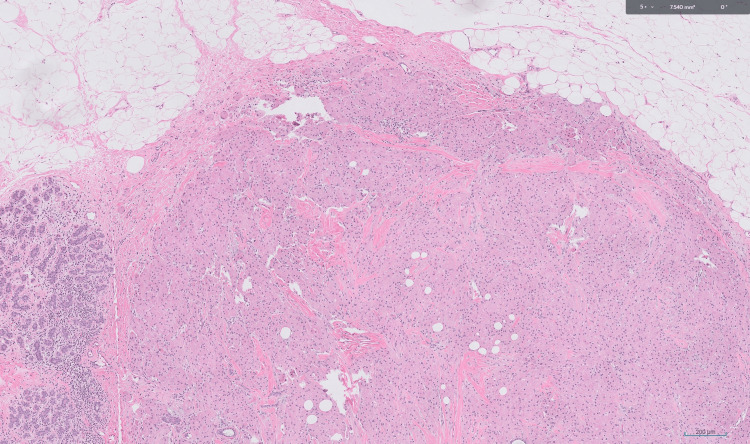
Granular cell tumor (x40 magnification) Tumor cells are polygonal with round nuclei, abundant eosinophilic granular cytoplasm.

**Figure 3:**
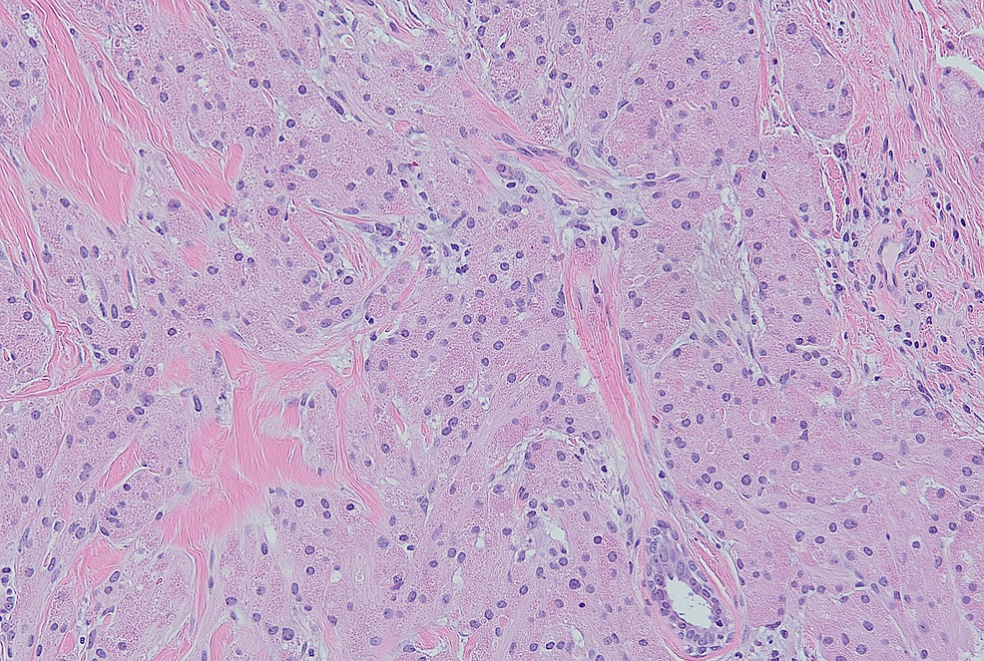
Granular cell tumor (x200 magnification) Epithelioid cells with round nuclei and abundant eosinophilic granular cytoplasm. Tumor cells show an infiltrative pattern.

Microscopic examination showcased breast parenchyma with terminal ductal units. Each terminal ductal unit maintained its lobular architecture, featuring two cell types lining without evidence of atypia or malignancy. Notably, a well-circumscribed lesion composed of monomorphic cells with pink granular cytoplasm and a low nuclear to cytoplasmic ratio was observed (Figures 1-3).

**Figure 4:**
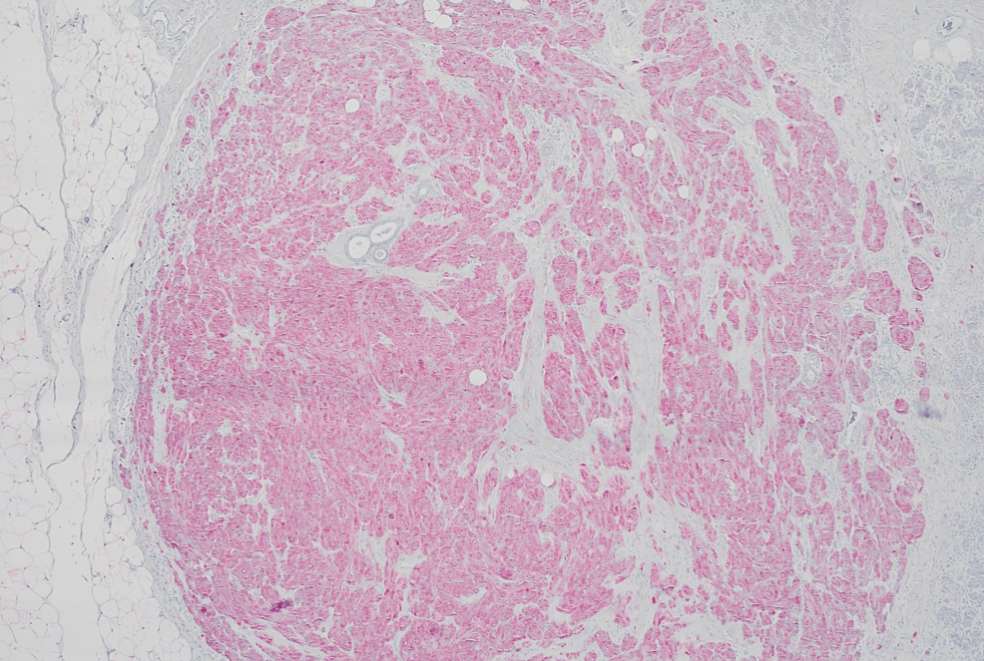
S100 immunohistochemistry (x20 magnification) Tumor cells stain for S100

IHC using S100 (Figure 3) and CD68 (Figure 4) stains confirmed the identity of these cells. 

**Figure 5:**
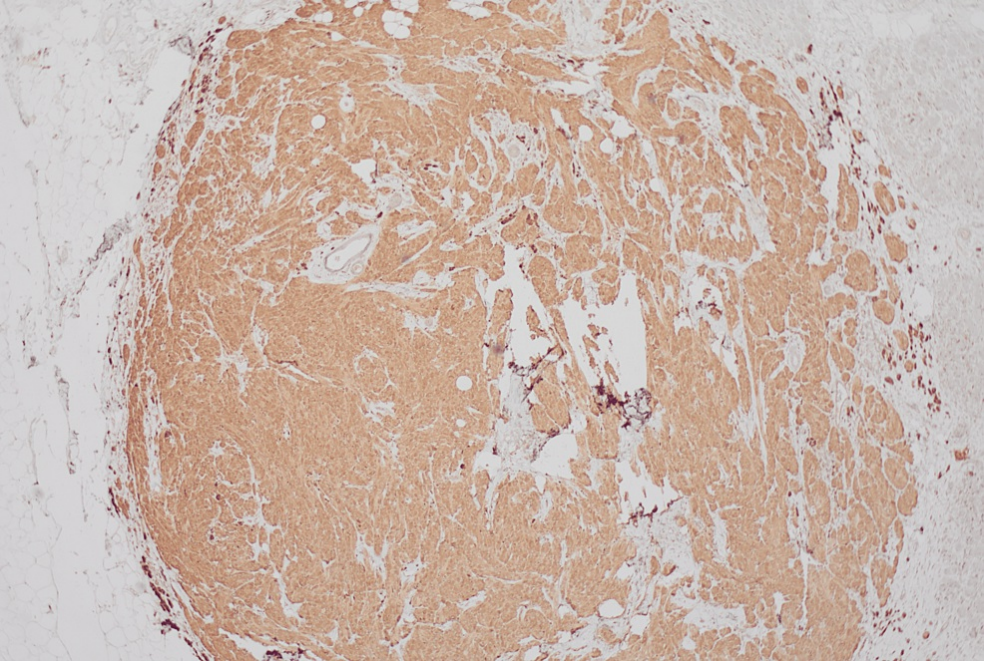
CD68 immunohistochemistry (x40 magnification) Tumor cells stain for CD68

The case was signed out as consistent with a benign GCT. There was no evidence of malignancy. Post-operative follow-up revealed a healing surgical site with no new masses, swelling, discharge, or skin changes.

This comprehensive diagnostic journey underscores the complexity of breast pathology and the importance of precise histopathological evaluation in arriving at an accurate diagnosis.

## Discussion

GCT is a benign neural tumor of neuroectodermal (Schwann cell origin) [2]. It is relatively rare and its usual site of occurrence include the skin, oral cavity, and subcutaneous tissue [2,3]. GCTs of the breast are very rare and account for 8.5 % of all GCTs, and like its counterpart at other sites of the body, GCT of the breast show similar morphology [1,3]. GCTs of the breast usually will not recur even with positive margins [4]. GCTs of the breast present a variable array of diagnostic imaging findings, often posing a challenge due to their potential mimicry of malignancy. Mammographically, these tumors typically manifest as small lesions, measuring less than 3 cm. The appearance can range from a round, well-circumscribed mass to an indistinct or spiculated lesion, making it difficult to distinguish from carcinoma.

On ultrasound, GCTs exhibit diverse characteristics, appearing as suspicious hypoechoic masses with irregular or poorly defined margins, often accompanied by marked posterior acoustic shadowing [5]. Alternatively, they may present as more benign-appearing, well-circumscribed solid masses. Variable vascularization and the presence of features such as a hyperechogenic halo and posterior acoustic shadowing depend on the degree of reactive fibrosis [5]. MRI reveals GCTs as low-to-intermediate signal intensity lesions on T1-weighted images, often inconspicuous on T2-weighted images. Contrast-enhanced MRI shows variable enhancement patterns following gadolinium administration, with both progressive (type 1) and wash-out (type 3) dynamic curves described [5]. Importantly, GCTs do not exhibit increased metabolic activity on PET-CT, providing a distinctive feature that aids in differentiation from malignancy. In summary, a comprehensive evaluation combining mammography, ultrasound, MRI, and PET-CT is crucial for accurate diagnosis, considering the diverse radiologic features exhibited by GCTs of the breast.

Histologically, GCT cells are usually in nests or sheets, growing in-between fibrous stroma of the breast. The cells are polygonal with abundant eosinophilic granular cytoplasm and have an oval-to-round nuclei. GCTs stain positively for S100 (alluding to its neuroectodermal origin), stains positive for lysosomal stain CD68, and is PAS/D (periodic acid-Schiff with diastase) positive in a coarse granular pattern [2]. Differentials for GCT include histiocytic type of lobular carcinoma. However, unlike GCT, this differential does not show the classic morphological appearance of GCT. Histiocytic variant of lobular carcinoma shows pleomorphic cells, is associated with an in-situ component, and is positive for cytokeratin. Both can be negative for p63 IHC stains. Another important differential is melanoma, which, like granular cells, stains S100. Melanoma usually presents as a lesion of the breast skin, which our patient did not present with, and it also shows involvement of the dermis and subcutaneous tissue. Melanoma would also show an in situ component and junctional activity. In addition to S100, melanoma cells would also stain for melanocytic markers such as SOX10 and HMB45 [6]. Our patient had the classic morphologic features for GCT and had no skin lesion that could be suggestive of melanoma. In addition, microscopic examination revealed no in situ or junctional component of melanoma.

Although GCTs are usually benign, there have been cases of malignant GCTs that show three or more of the following features: necrosis, vesicular nuclei with large nucleoli, pleomorphism, high nuclei to cytoplasmic ratio, spindle cell morphology, or any appreciable mitotic rate [2,4,6]. A tumor (GCT) with one or two of these features is classified as atypical or a tumor of uncertain malignant potential, warranting a close follow-up. Our case did not show any of the features listed above.

## Conclusions

By delving into the rare realm of breast GCTs through a comprehensive case study, we illuminate the intricate diagnostic challenges associated with this uncommon lesion. A meticulous histomorphological examination, complemented by judicious IHC, remains paramount in distinguishing the benign trajectory, as exemplified by our case lacking features indicative of malignancy. This diagnostic expedition not only contributes to the evolving landscape of breast pathology but also reinforces the indispensable role of histopathology in unraveling the complexities surrounding GCTs. It serves as a poignant reminder of the necessity for comprehensive diagnostic thoughts, embracing clinical acumen, radiological insights, and histopathological precision to navigate the nuances of rare breast lesions.
